# Beyond Pallidal or Subthalamic Deep Brain Stimulation to Treat Dystonia

**DOI:** 10.5334/tohm.935

**Published:** 2024-09-17

**Authors:** Vedant Garg, Venkat Srikar Lavu, Grace Hey, Brett Winter, Marcos Santana Firme, Justin D. Hilliard, Coralie De Hemptinne, Michael S. Okun, Joshua K. Wong

**Affiliations:** 1 Department of Neurology, Norman Fixel Institute for Neurological Diseases, University of Florida, Gainesville, FL, USA; 2 Department of Neurosurgery, Norman Fixel Institute for Neurological Diseases, University of Florida, Gainesville, FL, USA

**Keywords:** dystonia, neuromodulation, deep brain stimulation, thalamus, cerebellum

## Abstract

Deep brain stimulation of the subthalamic nucleus and globus pallidus internus is approved by the Food and Drug Administration for treating dystonia. Both targets have shown effectiveness in improving symptoms, but post-operative outcomes can vary significantly among patients. This variability has led researchers to explore alternative neuromodulation targets that might offer more consistent results. Emerging research has highlighted several promising new targets for DBS in dystonia. This review examines pre-clinical and clinical data on novel DBS targets for dystonia and explores non-invasive neuromodulation studies that shed light on the disease’s underlying pathological circuitry.

## A brief history of deep brain stimulation for dystonia

Dystonia is a movement disorder characterized by muscle contractions that result in abnormal postures, repetitive movements, or both [1]. It is the third most common movement disorder, with the prevalence of primary dystonia reported at 16.43 per 100,000 [2]. Although the exact pathophysiology of dystonia is not entirely understood, the basal ganglia cerebello thalamo cortical loop (BGCT) plays a central role in this disease and has revealed possible targets for surgical interventions, including deep brain stimulation (DBS) [3,4,5]. DBS is an effective neuromodulatory therapy that involves the implantation of electrodes for chronic stimulation of brain tissue. DBS is safe and effective in well-selected and appropriately screened patients [6].

In the early 1950s, lesional neurosurgery using radiofrequency ablation emerged as a therapeutic modality to treat dystonia. Various central nervous system structures were targeted, such as the medial thalamus, dentate nucleus, globus pallidus internus (GPi), subthalamic nucleus (STN) and other basal ganglia [7,8]. After noticing that unilateral pallidotomy improved the medication OFF state in people with Parkinson’s disease, pallidal lesions were used to treat primary generalized dystonia [4,9,10]. However, these lesions had significant limitations, particularly in treating axial symptoms with unilateral pallidotomy. Moreover, bilateral pallidotomy was linked to an increased risk of dysarthria, dysphagia, balance problems, and cognitive impairment [10,11].

**Figure 1:**
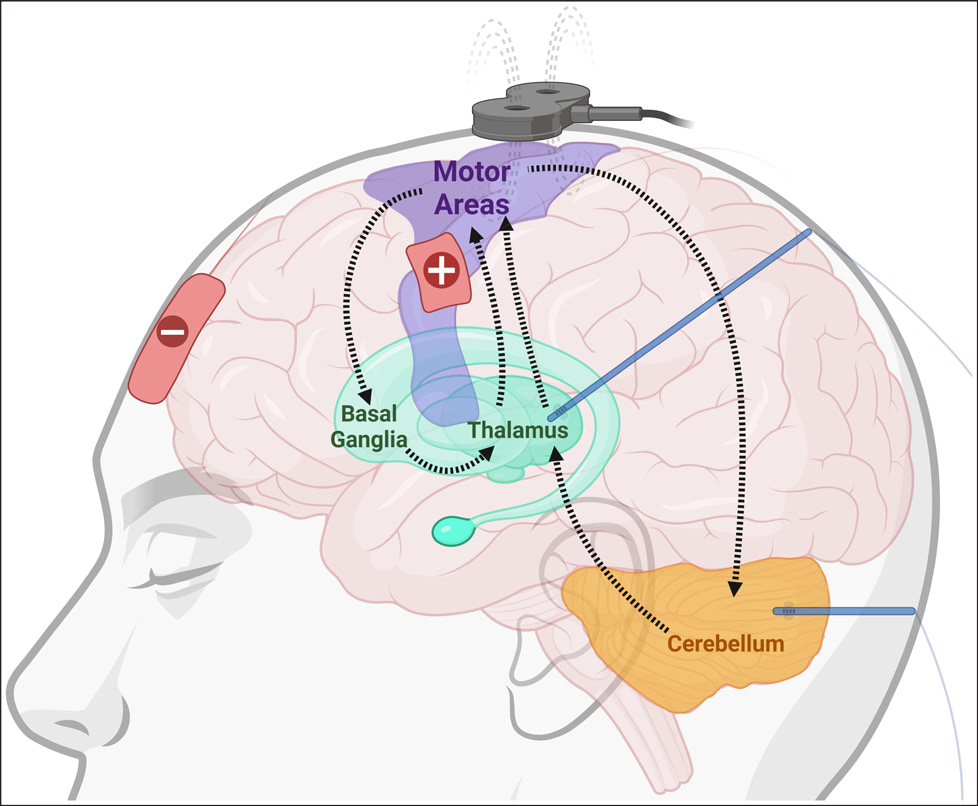
**The brain networks involved in dystonia**. The complex brain networks involved in the pathogenesis of dystonia are illustrated. These networks include the cerebello-thalamo-cortical network and basal ganglia-thalamo-cortical network. The ability to modulate these networks is also demonstrated here: transcranial direct current stimulation (red paddles), transcranial magnetic stimulation (black coil), and deep brain stimulation (blue leads).

**Figure 2:**
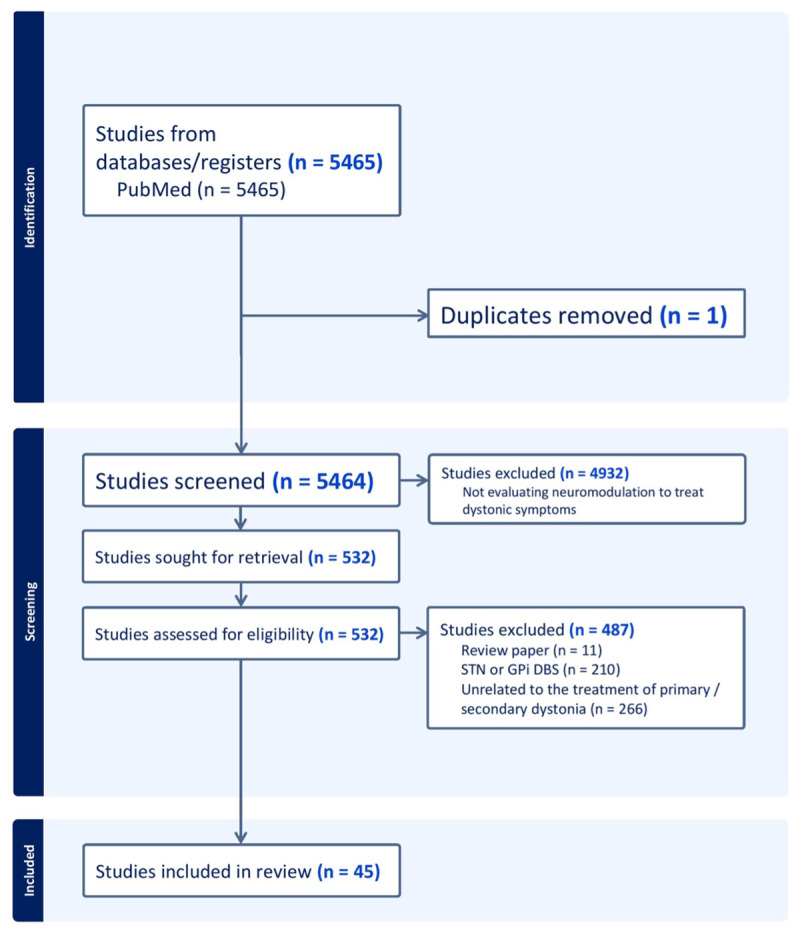
**PRISMA flow diagram outlining search strategy and screening process**. The process of our literature review, including the criteria for inclusion and exclusion, is depicted below. This study was conducted in accordance with the PRISMA guidelines.

In 1977, Mundiner first used DBS to treat cervical dystonia, achieving moderate success [4]. Following this, bilateral GPi DBS was investigated for primary generalized and segmental dystonia [12,13,14,15]. In 2003, these efforts led to the Food and Drug Administration granting a humanitarian device exemption for STN and GPi DBS to treat patients with chronic, medically intractable dystonia [9,16]. Although the GPi has been the main target for DBS in dystonia patients, other targets such as the ventral intermediate nucleus (VIM) and the STN have also been effective alternatives in specific cases [8,17,18,19]. As our understanding of dystonia pathogenesis progresses towards a network-based disease model, substantial evidence supports the utility of several new targets [8,20,21]. Here we define a network-based disease model as a condition where pathology or intervention in spatially distinct but interconnected brain regions impacts the same phenomenology, but in potentially different ways. Further, while GPi DBS can be effective for primary dystonias such as idiopathic or genetic dystonia, its efficacy on secondary dystonia such as post-stroke or tardive dystonia is less predictable, highlighting the need for alternative neuromodulation targets. In this review, we will explore the emerging and innovative targets for dystonia DBS beyond the already FDA-approved STN and GPi. We will also highlight noninvasive neuromodulation studies that have provided insight into the complex interplay of brain networks affected in dystonia (Figure 1). A complete review of the literature was undertaken using the PRISMA guidelines with the search strategy summarized in Figure 2 [22]. Our search terms can be seen in Supplemental Appendix 1.

## The cerebellum

Several animal studies have suggested that the cerebellum may be involved in the development of dystonia and the associated motor problems. DYT-TOR1 A is an inherited form of dystonia caused by a mutation in the TorsinA protein. Fremont et al. found that a lack of TorsinA in the cerebellum, rather than the basal ganglia, led to dystonia in a mouse model of DYT-TOR1 A [23]. They also observed abnormal cerebellar activity associated with dystonic symptoms, indicating the central role of the cerebellum in DYT-TOR1 A-related motor issues [23]. In rat models, injecting kainic acid into the cerebellar vermis resulted in dystonic reactions, with the severity depending on the dosage [24]. These kainic acid injections, an excitatory glutamate receptor agonist, into the cerebellar vermis also caused alterations in the basal ganglia, demonstrating an underlying network dysfunction seen in dystonia. LeDoux et al. further explored this by performing cerebellectomies (CBX) in a dystonic rat model and found that it eliminated dystonic motor signs and improved motor function compared to the preoperative state [25]. Overall these studies provide supporting evidence that cerebellar pathology contributes to the motor symptoms in dystonia.

Given the growing collection of basic science research supporting the involvement of the cerebellum in dystonia, multiple studies have investigated noninvasive cerebellar stimulation in humans. Bradnam et al. studied 8 patients with focal hand dystonia (FHD) treated with cerebellar transcranial direct current stimulation (tDCS) for 20 minutes at a constant current of 2 mA. The patients treated with anodal tDCS demonstrated improved performance in handwriting speed, pen pressure during handwriting, and circle drawing tasks [26]. The improvement of dystonic symptoms was also observed in cerebellar tDCS in two cases of cervical dystonia. Nguyen et al. described a 77-year-old woman with cervical dystonia who was previously treated with botulinum toxin injections and motor cortex 10 Hz repetitive transcranial magnetic stimulation (rTMS) with an incomplete response [27]. She was then treated with five sessions of cerebellum anodal tDCS and experienced a significant reduction in pain and dystonic symptoms with a 67%–91% reduction in TWSTRS scores. Her improvements were sustained for two months. These benefits were noted most prominently following right cerebellar hemisphere stimulation. Bradnam et al. described a similar case in which a 47-year-old woman with a 15-year history of cervical dystonia was treated with tDCS in the cerebellum and primary motor cortex for 12 weeks [28]. The patient experienced a 55% reduction in the TWSTRS pain score, a 40% reduction in the craniocervical dystonia questionnaire (CDQ-24) and a 39% reduction in the cervical dystonia impact profile (CDIP-58).

Two sham-controlled trials using rTMS demonstrated that cerebellar theta burst stimulation (TBS) could modulate neuroplasticity and improve dystonic symptoms in cervical dystonia [29,30]. Koch et al. tested the efficacy of continuous cerebellar TBS (pulse bursts every 200 ms for 40s) on 20 patients with cervical dystonia, while Bradnam et al. evaluated the effects of intermittent TBS (an interval pattern of a 2s TBS train repeated every 10 seconds) on 16 patients with cervical dystonia. Both studies found a statistically significant decrease in total TWSTRS score of almost 5 points following treatment with TBS. In a study conducted by Bologna et al., while continuous cerebellar TBS did not impact clinical outcomes in FHD and cervical dystonia patients, it reduced primary motor cortex excitability in cervical dystonia but not FHD, suggesting that the patterns of cerebellar dysfunction may vary based on the type of dystonia [31]. These non-invasive neuromodulatory studies collectively indicate that the cerebellum might play a key role in the network causing motor symptoms in dystonia. Therefore, targeting the cerebellum could be a promising area for future interventional studies.

Sokal et al. sought to explore this potential and conducted a study of 10 patients with focal or segmental dystonia secondary to cerebral palsy treated with deep anterior cerebellum stimulation (DACS). This involved the implantation of a quadripolar DBS electrode in the anterior lobe of the cerebellum. Longitudinal analysis ranging from 2–11 years after intervention revealed a significant reduction of upper extremity spasticity in 8 patients (p = 0.01), lower extremity spasticity in 7 patients (p = 0.02), and improved UDRS scores (p = 0.043) [32].

Horisawa et al. also described the use of cerebellar DBS in a case report of a 16-year-old girl with generalized fixed dystonia who had a minimal response to intrathecal baclofen therapy and bilateral pallidotomy. DBS targeting the bilateral superior cerebellar peduncles (SCPs) and dentate nuclei (DNs) was performed. The authors observed that SCP stimulation could relax muscles better than DN stimulation after two weeks of stimulation using amplitudes greater than 7V. Bilateral SCP DBS was programmed at an amplitude of 8V, a pulse width of 150 μsec, and a frequency of 200 Hz, which resulted in significant clinical improvement. The authors described the patient transforming from a preoperative bedridden state to eating and writing more independently after five months of DBS therapy [33]. Given the involvement of the SCP and DN in the dentate-thalamocortical pathway, stimulation may have modulated cortical excitability and potentially played a role in the clinical benefits [33]. These observations suggest that cerebellar DBS may also be effective in dystonia by modulating the pathologic connectivity between the cerebellum and the thalamocortical network.

In a study conducted by Cajigas et al., three patients with dyskinetic cerebral palsy, a subset of cerebral palsy highlighted by dystonia and choreoathetosis, were treated with bilateral cerebellar DBS targeting the DN and cerebellar outflow pathways. While all patients experienced a subjective improvement of motor function, the Burke-Fahn-Marsden Dystonia Rating Scale (BFMDRS) movement subscale response was variable, with an improvement of 19%–40% [34]. Given the damage to the basal ganglia and thalamic structures associated with cerebral palsy, the cerebellum may be an effective stimulation site for these scenarios. Currently, a series of double-blinded, randomized N-of-1 trials are being conducted to study quality of life and motor outcomes in 10 patients with dyskinetic cerebral palsy after being treated with bilateral cerebellar DBS of the DN and cerebellar outflow pathways [35].

## The thalamus

Multiple studies report using VIM DBS for dystonic tremor and have observed robust clinical outcomes. In a report by Coenen et al., a 37-year-old female with a 19-year history of dystonic head tremor experienced a significant improvement (>90%) in head tremor three months after bilateral VIM DBS [36]. Similarly, improvement of dystonic tremor and fine motor skills after bilateral VIM DBS was also observed in a 25-year-old man with dystonia-choreoathetosis due to cerebral palsy, with the BFMDRS motor score and disability score improving by 13 and 4 points, respectively [37]. Since the VIM receives input from the cerebellum, as fibers travel to the motor and premotor cortices, VIM DBS may effectively treat dystonic tremor by modulating the pathological activity of the cerebello-thalamo-cortical network.

In addition to the VIM, the ventralis oralis anterior (Voa) has also been explored as a DBS target for dystonia. In a study by Mongardi et al., three patients with dystonic tremor experienced clinical benefit after Voa DBS, with all three patients observing significant improvements in their Fahn Tolosa Marin Tremor Rating Scale (FTMTRS) and BFMDRS motor scores. In patients with both tremor and dystonic features, the authors proposed the Voa as a potential DBS target over the VIM or GPi, as the Voa functions as an intersection between the cerebellothalamic and pallidothalamic pathways [38].

The thalamus may also serve as a viable DBS target for dystonia especially after suboptimal response following GPi DBS. Ghika et al. described a 26-year-old patient with generalized post-anoxic dystonia who underwent bilateral Voa DBS after marginal improvement following six weeks of bilateral GPi DBS. At 4-month follow-up, the patient experienced a reduction in dystonic symptoms and was able to walk independently with a documented UDRS improvement of 50% [39]. While a significant change was observed following Voa DBS, the response duration to GPi DBS may in many cases be delayed weeks to months, complicating the interpretation of this and other similar dystonia DBS cases in which clinical failure may be prematurely concluded in GPi DBS. An interesting point to consider is that this case report described DBS of multiple interconnected structures and potentially related nodes within the underlying pathological network. Intervening at different points within this network may lead to variations in the response time for symptomatic improvement. Further studies are needed to elucidate if these brief reports represent a common pathway and if there are equivalent responses based on the target of intervention.

In addition to the traditional VIM and Ventralis-oralis (VO) targeting, blended targeting strategies have also been explored for DBS, particularly in writer’s cramp dystonia. Cho et al. describe a case study in which a 36-year-old female with focal hand dystonia presented with right-hand tremors and sensory abnormalities. After treatment with unilateral VO complex (Voa/Vo posterior) DBS, she experienced significant clinical improvement in the BFMDRS motor score which decreased from 4 to 1 after 12 months of therapy [40]. A similar improvement was described by Fukaya et al., where five patients with treatment-resistant idiopathic writer’s cramp underwent thalamic DBS, with four patients having one lead in the VO/VIM and one patient with a lead in the GPi and VO/VIM. After 24 months of stimulation, all patients experienced a significant improvement in handwriting with the mean BFMDRS handwriting score decreasing from 3.2 to 0.2 (p < 0.001) [41]. Interestingly, bipolar stimulation of the VIM and VO yielded the greatest clinical improvement when compared with monopolar stimulation of either the VO or VIM. In the patient with two DBS leads, thalamic stimulation was continued and chosen over GPi stimulation as it resulted in improved handwriting.

Thalamic DBS has also been reported to be beneficial in myoclonus dystonia. In a report of one myoclonus dystonia patient with double mutations in the DYT-TOR1 A and DYT11 genes, bilateral VIM DBS resulted in a BFMDRS motor score decreasing from 35 to 8, the BFMDRS disability score decreasing from 17 to 5, and the Unified Myoclonus Rating Scale rest/action sub_score decreasing from 24 to 0 after six months of therapy [42].

Various studies have described GPi and thalamic DBS to treat dystonia, suggesting a synergistic effect when applying co-stimulation. Slotty et al. reported results in a single patient with hemidystonia secondary to putaminal stroke after implantation with GPi and VIM/VO DBS. Neither of the targets resulted in an improvement in the BFMDRS when evaluated individually. However, simultaneous stimulation of the VIM/VO and GPi DBS leads resulted in an improvement in the BFMDRS by 25% and the 36-Item Short Form Health Survey by 86% [43]. Chang et al. described five patients with focal or segmental upper extremity dystonia who underwent unilateral GPi and VO DBS. When comparing benefit based on target choice, the authors found that stimulating both targets was more beneficial than stimulating either target in isolation [44]. This observation was also reported by Trompette et al., where three patients with dystonic tremor underwent bilateral GPi and VIM DBS. At 1-year post-implantation, both targets were evaluated in several ON-OFF permutations (e.g. GPi ON, VIM OFF). The mean total FTMTRS and BFMDRS scores improved maximally during simultaneous GPi and VIM DBS compared to either target alone [45].

Dual lead pallidal and thalamic DBS have also been demonstrated to provide long-term benefit in dystonia. Krause et al. describe six patients with myoclonus dystonia and bilateral VIM and GPi DBS and one myoclonus dystonia patient with bilateral VIM DBS across a mean follow-up of 12 ± 1.7 years (range 7–20 years). In this study, four patients remained on pallidal stimulation at long-term follow-up and experienced a mean reduction in myoclonus of 70% and a mean improvement in motor BFMDRS of 40%. Two patients remained on VIM and GPi stimulation at long-term follow-up and experienced a mean reduction of myoclonus by 61% on the Unified Myoclonus Rating Scale and a 17.9% motor BFMDRS improvement. The patient with bilateral VIM DBS experienced a 72% improvement in myoclonus and a motor BFMDRS improvement of 50% [46].

## The cortex

Researchers have also investigated noninvasive cortical-based neuromodulation techniques in dystonia cases. Although these methods affect neural tissues differently than DBS, they provide information on the dysfunctional brain networks underpinning dystonia. Insights from these studies could potentially guide the identification of new DBS targets and help fully map the brain regions involved in dystonia.

## Premotor cortex

Murase et al. compared the efficacy of subthreshold low frequency (0.2 Hz for 250 stimuli) rTMS delivered to the primary motor cortex, premotor cortex, and supplementary motor area in 9 patients with FHD. The greatest improvement in handwriting was reported in 7/9 patients who received premotor cortex stimulation, which in contrast to other targets, enhanced handwriting metrics, such as the mean tracking error from the target (p = 0.004) and pen pressure (p = 0.001). Additionally, it significantly prolonged cortical silent periods (p = 0.02), indicating stronger intracortical inhibition and a potential mechanism for intervention [47]. Alternative rTMS stimulation parameters applied to the premotor cortex were also effective in treating FHD [48,49].

The combined use of neuromodulation and motor training has been explored in treating focal hand dystonia, although with variable results. Souza et al. described a 46-year-old woman with FHD who experienced an improvement in dystonic symptoms after 15 sessions of rTMS of the premotor cortex followed by 30 minutes of kinesiotherapy, while Kimberley et al. reported that nine patients with FHD experienced no additional benefit of sensorimotor training when combined with rTMS of the premotor cortex [50,51].

In a randomized sham-controlled study, eight patients with cervical dystonia were treated with 15-minute sessions of low-frequency rTMS (0.2 Hz) over the anterior cingulate cortex, supplementary motor area, dorsal premotor cortex, and the primary motor cortex. While all sites except the anterior cingulate cortex demonstrated an improvement in TWSTRS severity scores, the greatest improvement was observed in the dorsal premotor cortex (2.9 ± 3.4) and the primary motor cortex (3.0 ± 4.8) [52]. A similar improvement of cervical dystonia after rTMS over the premotor cortex was noted in a case described by Allam et al. [53].

## Primary motor cortex

In two separate studies, the primary motor cortex was demonstrated to be effective in treating cervical dystonia. Richardson et al. reported that rTMS of the primary motor cortex in 8 cervical dystonia patients resulted in a TWSTRS improvement of 3.0 ± 4.8 [52]. When combined with kinesiotherapy, the primary motor cortex also served as an effective stimulation target for tDCS in cervical dystonia [50].

Using rTMS over the motor cortex has been observed to alleviate dystonic symptoms in FHD in both short and long-term treatment. Betti et al. described a 55-year-old man with FHD who experienced a significant improvement in motor coordination in a task resembling the dystonic-inducing symptoms and a reach-to-grasp task after five 30-minute sessions of 1 Hz rTMS [54]. Similarly, Siebner et al. found that 8/16 patients experienced a transient improvement in handwriting after 30 minutes of 1 Hz rTMS of the motor cortex [55]. Furukawa et al. sought to evaluate the immediate and long-term efficacy of rTMS. In a 40-year-old woman with FHD who was treated with 25 sessions of rTMS over one year and ten months, substantial improvements were observed with gradually lower numbers of stimulations throughout treatment [56]. In contrast with rTMS studies, Benninger et al. found no significant benefit to tDCS of the primary motor cortex in patients with FHD [57].

In a randomized study, 60 Wilson’s disease patients with upper limb dystonia received 30-minute 10 Hz rTMS sessions over the primary motor cortex or sham stimulation for seven consecutive days. After the 7th session, two weeks, and four weeks, the muscle tension and stiffness, the Modified Ashworth Scale (MAS), the Unified Wilson’s Disease Rating Scale (UWDRS), and the BFM in the treatment group were significantly lower than the control group (p < 0.01 for all) [58]. While high-frequency rTMS of the motor cortex appeared to alleviate symptoms of upper limb dystonia, Sharma et al. presented a case in which low-frequency rTMS failed to effectively treat dystonic symptoms. Although the patient tolerated the treatment well, he experienced no improvement in dystonic symptoms based on video analysis, Clinical Global Impression, and PHQ-9 scores [59].

## The future of DBS for dystonia

DBS can be an effective therapy for select patients with dystonia refractory to medication. While the GPi remains the most common DBS target for the treatment of dystonia, the clinical outcomes can be variable and the programming optimization process is often challenging with benefits noted in a delayed fashion. There has been emerging evidence to suggest several novel targets may be viable for neuromodulatory therapy (Table 1). Although dystonia has historically been understood to be associated with local basal ganglia dysfunction, newer hypotheses have reframed dystonia as a network disorder that involves the basal ganglia-thalamo-cortical circuit with a significant contribution from the cerebellum [32,33]. There are multiple animal models demonstrating the involvement of the cerebellum in the pathogenesis and motor dysfunction observed in dystonia, and both noninvasive cerebellar stimulation techniques including tDCS and rTMS along with invasive methods such as DBS and DACS have been found to effectively reduce dystonic symptoms. Although further studies will be needed to fully elucidate the mechanism and outcomes of cerebellar DBS in dystonia, there is growing optimism for utilizing this target.

**Table 1:** Overview of select studies demonstrating potential DBS targets for treating dystonia.


CEREBELLUM	

Fremont et al, Alvarez et al, LeDoux et al [23,24,25]	Mouse and rat models have suggested the involvement of the cerebellum in the pathogenesis and motor dysfunction seen in dystonia.

Bradnam et al, Nguyen et al, Bradnam et al [26,27,28]	Cerebellar tDCS improved handwriting metrics in patients with focal hand dystonia and dystonic symptoms and pain in patients with cervical dystonia.

Koch et al, Bradnam et al, Bologna et al [29,30,31]	Continuous and intermittent theta burst stimulation using rTMS significantly decreased TWSTRS scores in cervical dystonia patients, demonstrating its ability to modulate neuroplasticity and improve dystonic symptoms. Furthermore, patterns of cerebellar dysfunction may vary based on the type of dystonia.

Sokal et al. [32]	Deep anterior cerebellum stimulation significantly reduced upper and lower extremity spasticity and improved UDRS scores in patients with focal or segmental dystonia secondary to cerebral palsy.

Horisawa et al. [33]	In a patient with generalized fixed dystonia, cerebellar DBS resulted in a significant clinical improvement.

Cajigas et al, San Luciano et al [34,35]	In 3 patients with dyskinetic cerebral palsy, bilateral cerebellar DBS targeting the DN and cerebellar outflow pathways produced subjective and objective motor function improvements.

**THALAMUS**	

Coenen et al, Wolf et al [36,37]	In patients with dystonic head tremor and dystonia-choreoathetosis due to cerebral palsy, VIM DBS significantly improved dystonic tremor.

Mongardi et al. [38]	Patients with dystonic tremor experienced improvements in their FTMTRS and BFMDRS motor scores after Voa DBS.

Ghika et al. [39]	After a suboptimal response from GPi DBS, a patient with generalized post-anoxic dystonia underwent bilateral Voa DBS that resulted in a reduction of dystonic symptoms and UDRS improvement of 50%.

Cho et al, Fukaya et al [40,41]	In patients with writer’s cramp dystonia, a blended strategy targeting the VIM and VO resulted in the greatest improvement of BFMDRS motor and handwriting scores.

Wang et al. [60]	In a myoclonus dystonia patient with double mutations in the DYT-TOR1 A and DYT11 genes, thalamic DBS significantly improved BFMDRS motor and disability scores and the Unified Myoclonus Rating Scale rest/action subscore.

**MULTIPLE TARGETS**	

Slotty et al, Chang et al, Trompette et al [43,44,45]	In patients with hemidystonia, focal or segmental upper extremity dystonia, and dystonic tremor, dual lead pallidal and thalamic DBS resulted in greater benefit than when applying stimulation of either region individually.

Krause et al [46]	Patients with myoclonus dystonia and pallidal and thalamic DBS, pallidal DBS only, and thalamic DBS only saw a reduction of myoclonus of 61.8%–72% and an improvement in motor BFMDRS of 17.9%–50% across a mean follow up of 12 ± 1.7 years (range 7–20 years).

**CORTEX**	

Murase et al, Kimberley et al, Borich et al [47,49,51]	In patients with focal hand dystonia, premotor cortex rTMS improved handwriting metrics and prolonged cortical silent periods.

Souza et al, Kimberley et al [50,51]	In patients with focal hand dystonia, the use of rTMS of the premotor cortex in combination with motor training has produced variable results.

Richardson et al, Allam et al [52,53]	In patients with cervical dystonia, rTMS of the premotor cortex resulted in a significant clinical improvement, especially compared to other stimulation sites.

Richardson et al, Souza et al [50,52]	Both rTMS and tDCS of the primary motor cortex were effective targets for treating cervical dystonia.

Betti et al, Siebner et al, Furukawa et al, Benninger et al [54,55,56,57]	While rTMS of the motor cortex alleviated dystonic symptoms in focal hand dystonia in both short and long-term treatment, tDCS of the primary motor cortex resulted in no significant improvement in focal hand dystonia.

Hao et al, Sharma et al [58,59]	While high-frequency rTMS of the motor cortex was able to alleviate symptoms of upper limb dystonia, low-frequency rTMS was unable to produce an improvement.


The thalamus plays an important role in motor function, given its key involvement in the basal ganglia-cerebello-thalamo-cortical circuit. Thalamic stimulation, including that of the VIM, Vop, Voa, and VLp, has been reported to be effective at improving dystonic tremor and other symptoms, especially when combined with pallidal stimulation. Specifically, we highlight observations from VIM and Voa thalamic modulation as these two sub-nuclei have been the most studied to date. The VIM receives input from the cerebellum via the dentato-rubral-thalamic tract and has been implicated in treating dystonic tremor. Furthermore, the impact of VIM DBS on dystonic tremor and the proximity of the VIM to cerebellar outflow pathways also highlight the role that the cerebellum might play in motor dysfunction in dystonia. Voa DBS has demonstrated some effectiveness in alleviating dystonic tremor and possibly other dystonic symptoms. Its impact has been attributed to its role as a node within the pallido-thalamic pathway. Combined pallidal and thalamic stimulation may synergistically affect dystonic symptoms, suggesting the potential presence of overlapping pathology of the cerebello-thalamo-cortical network in concert with the classical basal-ganglia-thalamo-cortical network.

As we consider expanding our framework of the pathological networks associated with dystonia, we highlight several studies in this review that demonstrate transient improvement in dystonia symptoms spanning from noninvasive cortical neuromodulation. These studies provide supporting evidence for the involvement of a broader network underpinning dystonia. This network remains to be fully characterized. However, as we deepen our understanding of this network, it may lead to a convergence of information for refining neuromodulation in existing DBS targets or proposing novel locations for intervention. Novel brain targets for the treatment of dystonia may hold value beyond neurotherapeutics, as their reports fill in missing pieces to the pathophysiological puzzle.

## Supplemental Appendix 1

### PubMed MESH search terms

((((((dystonia) AND (transcranial magnetic stimulation OR deep brain stimulation OR transcranial direct current stimulation OR transcranial alternating current stimulation OR neuromodulation))) NOT (Review[Publication Type])) NOT (Meta-Analysis[Publication Type])) NOT (Systematic Review[Publication Type])) AND (english[Language]).

Produced 5465 results on PubMed.
